# Encapsulation of *Bacillus subtilis* in Chitosan Gel Beads for Eco-Friendly Crop Protection

**DOI:** 10.3390/gels11040302

**Published:** 2025-04-19

**Authors:** Vladimir Krastev, Nikoleta Stoyanova, Iliyana Valcheva, Donka Draganova, Mariya Spasova, Olya Stoilova

**Affiliations:** 1 Laboratory of Bioactive Polymers, Institute of Polymers, Bulgarian Academy of Sciences, Akad. G. Bonchev St., bl. 103A, 1113 Sofia, Bulgaria; v_krastev@polymer.bas.bg (V.K.); nstoyanova@polymer.bas.bg (N.S.); mspasova@polymer.bas.bg (M.S.); 2 Biodinamika Ltd., 4000 Plovdiv, Bulgaria; valchevailiana1@gmail.com (I.V.); donkadraganova@gmail.com (D.D.)

**Keywords:** chitosan gel beads, *Bacillus subtilis*, encapsulation, biocontrol, plant protection

## Abstract

Chitosan gel beads represent a promising biopolymer-based delivery system for encapsulating *Bacillus subtilis*, an effective biocontrol agent in sustainable agriculture. This study investigates the influence of chitosan molecular weight on bead structure, water retention, and swelling behavior, as well as its impact on the viability and antifungal activity of encapsulated *B. subtilis*. The results demonstrate that chitosan provides a protective microenvironment, enhancing microbial viability, promoting colonization, and ensuring controlled release for prolonged plant protection. Moreover, encapsulation within chitosan gel beads preserved bacterial viability during long-term storage for up to 90 and 180 days. Additionally, the biodegradable and antimicrobial properties of chitosan contribute to pathogen suppression while supporting the plant growth-promoting activities of *B. subtilis*. The encapsulated bioagent exhibited strong antifungal activity against *Fusarium avenaceum* and *Rhizoctonia solani*, highlighting the effectiveness of this eco-friendly approach as an alternative to chemical pesticides. These findings underscore the potential of chitosan-based formulations to enhance the efficacy of bioinoculants, offering a sustainable solution for modern crop management.

## 1. Introduction

The increasing demand for sustainable agricultural practices has intensified efforts to develop eco-friendly alternatives to chemical pesticides. Among these alternatives, biological control agents (bioagents) have gained significant attention thanks to their ability to suppress phytopathogens, stimulate plant growth, and enhance soil health while minimizing environmental impact.

*Bacillus subtilis* is a well-established biocontrol agent known for its production of a diverse array of antimicrobial compounds, its ability to elicit plant defense mechanisms, and its role in promoting plant growth [1,2,3]. Specifically, *B. subtilis* suppresses pathogen proliferation through competition for nutrients and space, the production of antifungal lipopeptides, and induction of systemic resistance in plants. Additionally, its ability to form resilient endospores enhances its persistence in agricultural environments. However, the successful field application of microbial bioagents in agriculture remains challenging. The primary obstacles include the susceptibility of microbial bioagents to environmental stressors, reduced survival in soil, and difficulties in maintaining prolonged activity after application [1].

To overcome these limitations, encapsulation technology has emerged as an effective approach to protect microbial bioagents during storage and field application while ensuring their sustained release and prolonged biocontrol activity [4,5,6,7]. Encapsulation within an appropriate polymer matrix shields microorganisms from environmental stressors such as temperature fluctuations, moisture variations, and pH changes, thereby enhancing their viability and efficacy over time [8,9,10]. Additionally, controlled-release mechanisms reduce the need for frequent applications, improving the sustainability of biocontrol measures. Various natural polymers, including chitosan, cellulose, alginate, and gelatin, have been employed for microbial encapsulation, offering protection against both abiotic and biotic stress while preserving metabolic activity [11]. Notably, certain polymer carriers not only function as protective matrices but also serve as nutrient sources, fostering microbial growth and promoting plant health.

Chitosan, a natural, biodegradable, and biocompatible polysaccharide derived from chitin, has emerged as a promising candidate for microbial encapsulation. Its inherent antimicrobial properties, coupled with its ability to form gels and beads, make it highly suitable for developing biocontrol formulations [12,13,14,15]. Chitosan exhibits significant antifungal activity against a broad spectrum of phytopathogenic fungi. Studies have shown that it effectively inhibits the growth of *Aspergillus ochraceus* by disrupting cell morphology and downregulating genes involved in ribosome biogenesis, which are essential for protein synthesis [16]. In agricultural applications, chitosan has been widely used to control postharvest diseases. For example, water-soluble chitosan significantly inhibited *Colletotrichum capsici*, the causative agent of anthracnose in chili peppers, by preventing spore germination and mycelial growth [17]. The molecular weight of chitosan plays a crucial role in its antifungal activity. Low molecular weight chitosan has demonstrated potent antifungal effects against clinical isolates of *Candida spp*., including fluconazole-resistant strains, particularly under acidic conditions. In contrast, high molecular weight chitosan has been effective in reducing anthracnose incidence in papaya fruits by enhancing the synthesis of ascorbic acid and phenolic compounds, thereby boosting the fruit’s antioxidant capacity [18,19]. Another important parameter influencing the antifungal activity of chitosan is its degree of deacetylation (DD). Several studies have reported a positive correlation between DD and antifungal efficacy. This effect is attributed to stronger electrostatic interactions between the protonated amine groups of chitosan and the negatively charged components of fungal cell membranes. These interactions occur in acidic environments, where the pH is lower than the protonation constant (pKa) of the specific chitosan sample. The number of protonated amine groups is directly related to the degree of deacetylation. Moreover, the influence of DD may partially overlap with the effects of molecular weight [20]. For instance, the antifungal activity of chitosan with molecular weights ranging from 42.5 to 135 kDa and DD from 39% to 98% was tested against *Aspergillus niger*, *Fusarium oxysporum*, and *Alternaria solani* [21]. A response was observed in all species, but it was strongly species-dependent. The growth inhibition of *A. solani* and *F. oxysporum* clearly correlated with chitosan DD, with higher DD values exhibiting stronger antifungal activity. In the case of *F. oxysporum*, the inhibitory effect was primarily influenced by molecular weight, although a DD above 59% was also necessary to significantly restrict growth. *A. niger* growth was inhibited by chitosan in general, but no clear influence of DD or molecular weight was observed.

Chitosan’s antifungal mechanism is multifaceted, involving interactions with fungal cell membranes that increase permeability, disrupt nutrient flow, and inhibit spore germination. Additionally, chitosan can induce the production of defense-related enzymes and phenolic compounds in plants, enhancing resistance against fungal infections. Incorporating chitosan into nanoparticle formulations has further improved its antifungal efficacy. Chitosan nanoparticles have been shown to inhibit the growth of phytopathogenic fungi such as *Fusarium verticillioides*, *Alternaria alternata*, and *Macrophomina phaseolina*, causing structural distortions in fungal hyphae and reproductive structures [22]. Chitosan gel beads, in particular, provide an optimal encapsulation matrix for biocontrol agents by maintaining a hydrated microenvironment that supports microbial viability while enabling the controlled release of bacterial spores upon application [23,24]. Additionally, chitosan can serve as a carbon and nitrogen source for encapsulated microorganisms, promoting their proliferation and enhancing biocontrol efficacy [25]. Previous studies have demonstrated the suitability of chitosan-based immobilization for *Trichoderma viride*, highlighting its potential for developing ecologically safe phytosanitary preparations [26]. The molecular weight of chitosan is an important factor in determining its physicochemical properties, such as viscosity, water retention, and swelling behavior. These properties, in turn, influence microbial encapsulation efficiency, controlled release, and the structural integrity of the delivery system [27]. However, only a few studies have examined how chitosan molecular weight affects the encapsulation and biocontrol performance of *Bacillus subtilis* [28,29].

This study aims to study the impact of chitosan molecular weight on the encapsulation, viability, and biocontrol potential of *B. subtilis* against fungal phytopathogens. The ability of chitosan beads to support microbial growth, enhance antifungal activity, and provide an eco-friendly alternative to chemical fungicides is evaluated. Viability tests showed that bacteria encapsulated within chitosan gel beads remained viable both after incorporation and during long-term storage for up to 90 and 180 days. These findings contribute to the development of sustainable biocontrol formulations with improved stability, effectiveness, and prolonged plant protection, reducing the reliance on chemical pesticides in modern agriculture.

## 2. Results and Discussion

The current trend in the development of environmentally friendly plant protection agents is based on the use of beneficial microorganisms, thus preserving the environment and overcoming the harmful effects of widely used pesticides. A major problem in the direct application of beneficial microorganisms is the reduction of their effectiveness during prolonged storage or when applied to untreated soils. Therefore, the aim of this study was to obtain chitosan gel beads as carriers for the beneficial microorganism *Bacillus subtilis* and to demonstrate their effectiveness as promising materials for biological plant protection against pathogenic microorganisms. Furthermore, the influence of the molecular weight of the chitosan used on the growth and biological activity of the beneficial microorganism encapsulated in the gel beads was also studied.

### 2.1. Chitosan Gel Bead Formation and Characterization

Chitosan gel beads were obtained using the simple coacervation method through capillary extrusion of a chitosan solution into an alkaline bath, as previously described [30]. Depending on the molecular weight of chitosan, solutions were prepared with the following concentrations: CS-HMW—1.5% in acetic acid solution; CS-MMW—2% in acetic acid solution; CS-LMW—2% in acetic acid solution; and COS—10% in aqueous solution. These specific concentrations of chitosan solutions were selected based on preliminary experiments aimed at identifying the optimal conditions for bead formation and stability for each molecular weight. The formation of the gel beads occurs due to the coacervation of the chitosan solution in the precipitating bath, which results from the conversion of the soluble cationic ammonium form (–NH_3_⁺) into the insoluble neutral amine form (–NH_2_) of chitosan. A part of the as-prepared chitosan gel beads was dried to a constant weight, while the rest were kept in water. For clarity, the gel beads before drying will be referred to as “coacervate”, whereas those that have been dried and subsequently swollen will be referred to as “dry”.

**Figure 1:**
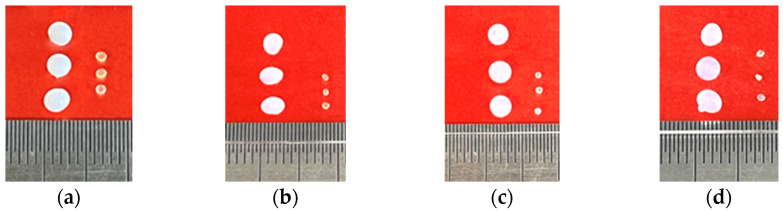
Coacervate (left) and dry (right) chitosan beads: (**a**) COS; (**b**) CS-LMW; (**c**) CS-MMW; (**d**) CS-HMW.

It was observed that, after drying, the coacervate beads significantly decreased in size (Figure 1). In addition, the coacervate beads were supple and white, whereas the dry beads became transparent and rigid. Table 1 presents the average weight and diameter of the coacervate and dry chitosan beads. As observed, the average weight and diameter of the beads significantly decreased after drying due to water loss, regardless of the type of chitosan used. However, the greatest weight loss and reduction in diameter were observed in CS-HMW beads. This was expected because chitosan with a higher molecular weight has longer polymer chains, leading to stronger intermolecular interactions and a greater ability to retain water in the coacervate state. Additionally, CS-HMW solutions tend to be more viscous due to extensive hydrogen bonding and entanglements between polymer chains, resulting in a more expanded structure in the hydrated state, which collapses more drastically upon water loss during drying. Therefore, the higher molecular weight of chitosan enhances water retention and structural expansion in the hydrated state, but upon drying, it leads to greater shrinkage and weight loss due to stronger polymer interactions and water-dependent swelling. This behavior can be linked to the structural changes observed in chitosan beads with varying molecular weight.

Similarly, the swelling degree of chitosan beads was found to be pH-dependent (Table 2), primarily due to its ionization behavior, which also affects the water retention capacity. Based on its pKa value (pKa = 6.5 [31]), the proportion of ionized chitosan (protonated –NH_2_ groups) was determined. In an acidic medium (pH 4), nearly all amino groups are protonated, whereas at pH 7, approximately 11% remain protonated, and at pH 9, only 0.3%. Under acidic conditions (pH < 7), chitosan dry beads undergo significant swelling and dissolve completely within 5 to 10 min. Interestingly, as the pH increases, the swelling degree of the chitosan dry beads also increases (Table 2). At a lower pH (around 4), chitosan amino groups are largely protonated, reducing intermolecular hydrogen bonding and facilitating greater swelling. As the pH increases, the degree of protonation decreases, and electrostatic repulsion between deprotonated groups enhances the swelling behavior. This interaction, coupled with the water retention properties of chitosan, suggests that both the molecular weight and the ionization state of chitosan influence its structural behavior in the hydrated state. Consequently, while higher molecular weight chitosan beads exhibit greater shrinkage upon drying, the swelling behavior at different pH values reflects the complex interplay of protonation, water retention, and intermolecular interactions.

Clearly, the swelling degree of chitosan beads also depends on the molecular weight of the chitosan used (Table 2). As observed, an increase in molecular weight leads to a higher swelling degree, regardless of pH. Higher molecular weight chitosan (i.e., longer polymer chains) results in greater chain entanglement, increasing intermolecular interactions and reducing dissolution. Moreover, the increased chitosan molecular weight facilitates water diffusion into the beads, resulting in a higher degree of swelling. Additionally, beads with higher chitosan content contain a greater number of –NH_2_ groups, which can be protonated to –NH_3_⁺, further contributing to swelling.

**Figure 2:**
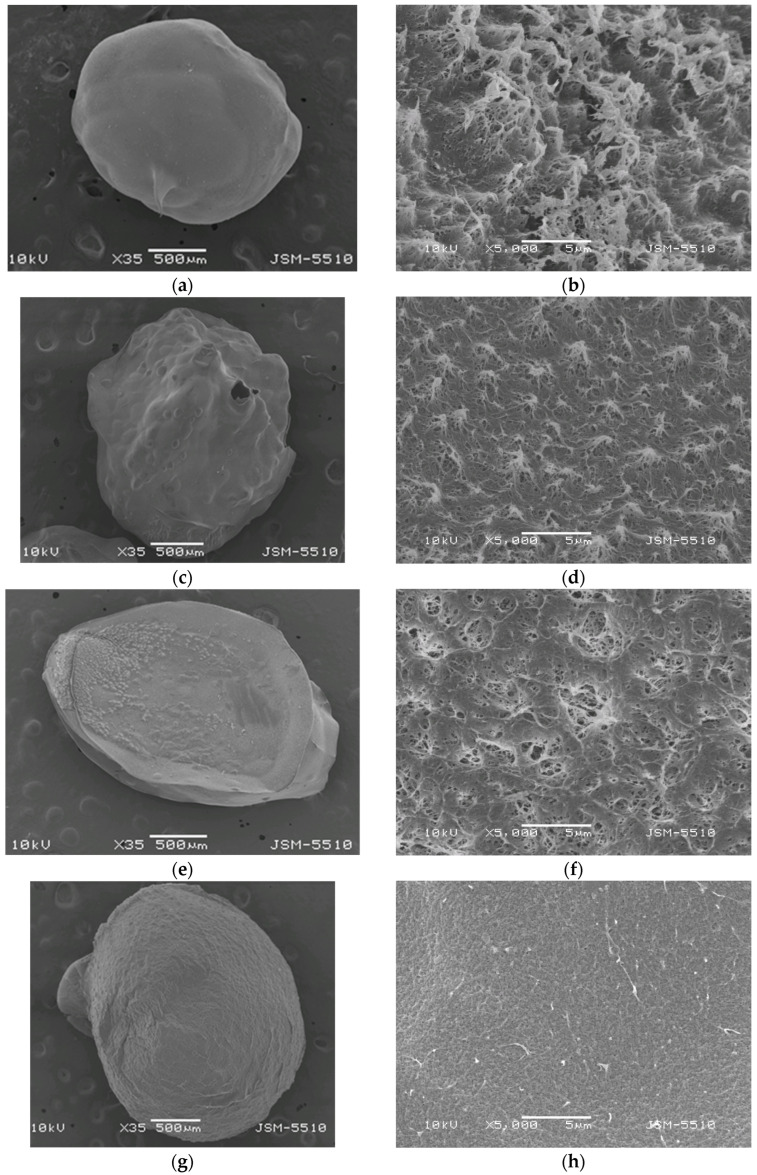
SEM images of freeze-dried coacervate gel beads of: (**a**,**b**) COS; (**c**,**d**) CS-LMW; (**e**,**f**) CS-MMW; (**g**,**h**) CS-HMW.

The differences in structural morphology, shape, and size among COS, CS-LMW, CS-MMW, and CS-HMW beads are further supported by the SEM images (Figure 2). Various magnifications were applied to the freeze-dried beads to examine both their outer and interior structures. As shown, all the chitosan beads were predominantly spherical or slightly oval in shape (Figure 2a–g). Beads obtained through capillary extrusion into a precipitation bath and subsequent freeze-drying exhibited a highly filamentous, porous structure. Their surfaces were rough, rubbery, fibrous, and folded, with visible wrinkles (Figure 2b–h). The interior of the beads contained micropores and microtube-like spaces, confirming their highly porous nature, despite their solid external appearance. Notably, the pore size decreased with the increasing molecular weight of chitosan, as the higher molecular weight chitosan beads exhibited a denser, more compact structure, with fewer and smaller pores. This suggests that as the molecular weight increases, the polymer chains become more entangled, reducing the formation of larger pores and promoting a tighter network structure. The exception observed for CS-MMW beads (Figure 2f) may be related to differences in viscosity or specific interactions during bead formation, which can influence the gel structure, independently of molecular weight.

### 2.2. Encapsulation of Bacillus subtilis in Chitosan Gel Beads

**Figure 3:**
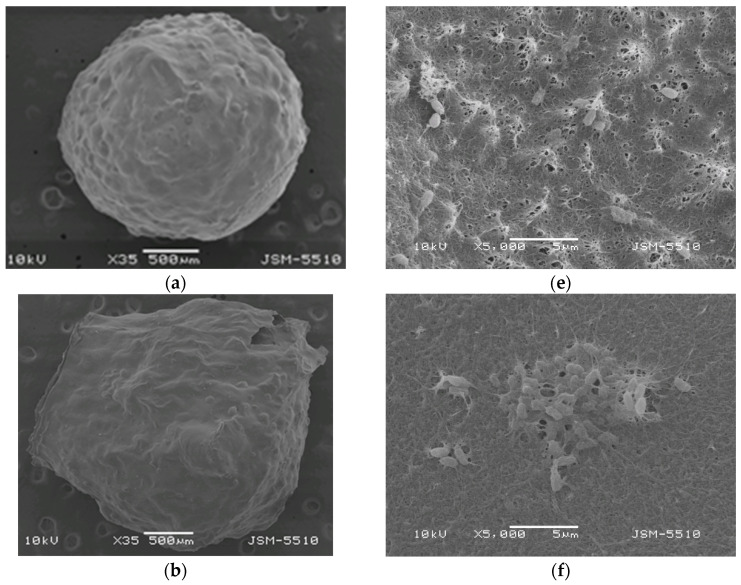

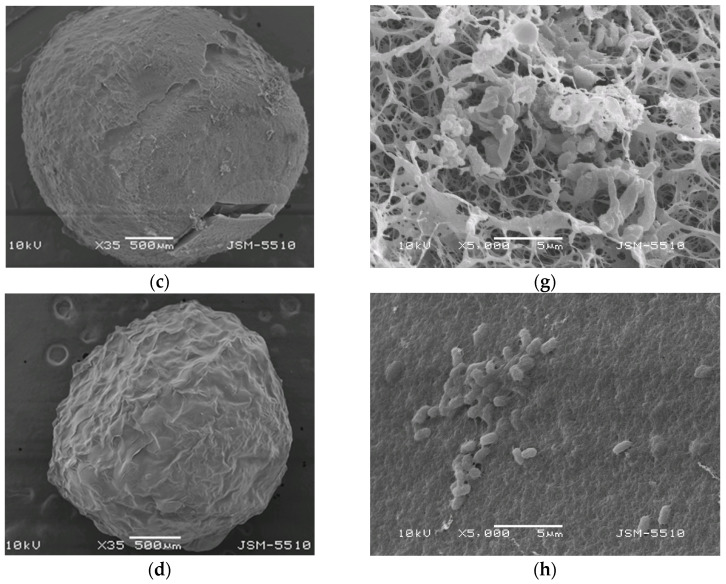
SEM images of freeze-dried coacervate gel beads of: (**a**,**e**) COS/*B. subtilis*; (**b**,**f**) CS-LMW/*B. subtilis*; (**c**,**g**) CS-MMW/*B. subtilis*; (**d**,**h**) CS-HMW/*B. subtilis*.

The next step involved the encapsulation of the bioagent into chitosan gel beads. In order to prepare an appropriate and eco-friendly biocontrol formulation, *Bacillus subtilis* was used as the bioagent. A suspension of *Bacillus subtilis* was added to chitosan solutions of varying molecular weights. The resulting mixtures were then extruded into an alkaline bath to form gel beads, which were subsequently washed until a neutral pH was reached. The surface morphology of the obtained chitosan beads with encapsulated *B. subtilis*, after freeze-drying, is shown in Figure 3. As seen, the beads preserve their spherical shape (Figure 3a–d). At higher magnification, the spores of *B. subtilis* are clearly observed, confirming the successful encapsulation (Figure 3e–h). The typical bead structure, which contains micropores and microtube-like spaces despite its solid external appearance, is preserved after encapsulation.

It is important to note that the molecular weight of chitosan also plays a role in the encapsulation process. Higher molecular weight chitosan (CS-HMW) beads tend to form a more compact structure with smaller pores, which could potentially affect the encapsulation efficiency. The denser structure may limit the accessibility of the chitosan matrix for the bioagent, whereas lower molecular weight chitosans forms larger, more porous beads, allowing for more efficient encapsulation of *B. subtilis* spores. This suggests that the choice of chitosan molecular weight influences both the morphology of the beads and the encapsulation efficiency of bioagents.

In order to demonstrate that *B. subtilis* spores were effectively encapsulated within the chitosan beads using the extrusion method, we performed cross-sectional SEM analysis (Figure S1). The images clearly reveal that the internal morphology of the beads is preserved, exhibiting a network of micropores and microtube-like structures, which confirms their highly porous nature despite a solid external appearance. Furthermore, the presence of *Bacillus subtilis* spores embedded within these internal structures confirms successful encapsulation rather than simple surface adsorption. These findings support the conclusion that the extrusion technique allows for uniform incorporation of the bacterial spores throughout the chitosan matrix. The porous architecture likely facilitates this distribution and may play a role in protecting the bacteria while enabling their gradual release or interaction with the surrounding environment. This structural insight strengthens the rationale for using such chitosan-based systems in microbial delivery applications.

### 2.3. Viability Assessment of B. subtilis Encapsulated in Chitosan Gel Beads

**Figure 4:**
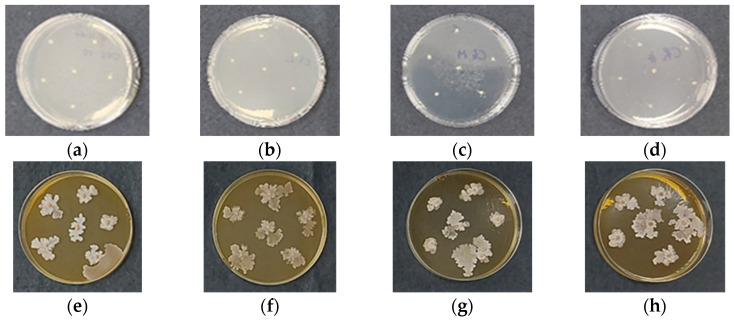
Digital images of chitosan gel beads after 48 h of incubation: (**a**) COS, (**b**) CS-LMW, (**c**) CS-MMW, (**d**) CS-HMW, (**e**) COS/*B. subtilis*, (**f**) CS-LMW/*B. subtilis*, (**g**) CS-MMW/*B. subtilis* and (**h**) CS-HMW/*B. subtilis*. Panels (**a**–**d**) represent the biological purity test, while panels (**e**–**h**) correspond to the viability test.

The viability of *Bacillus subtilis* to develop and reproduce after encapsulation in chitosan gel beads was evaluated. Control chitosan gel beads, without encapsulated bacterial spores, were tested for biological purity. As shown in the images taken at 48 h after bead inoculation (Figure 4), *B. subtilis* developed normally. The spores encapsulated in the coacervate gel beads initiated their development without delay. After 48 h, differences in growth rate and variations in the morphological structure of the formed bacterial colony were observed. At the end of the test period (Figure 4e–h), a significant difference in biomass appearance became evident. Microscopic observations confirmed that the spores from these beads had been activated, completed their vegetative development, and formed new spores. The control group (Figure 4a–d) demonstrated that the chitosan did not interfere with sporulation, and the gel beads remained biologically pure, with no development of accompanying microflora. Therefore, the spores encapsulated in the chitosan gel beads remain viable, and *B. subtilis* retains its ability to develop and reproduce normally. These results indicate that the developed biohybrid chitosan gel beads are promising formulations for the biological protection of plants against pathogenic microorganisms.

It is also important to note that the molecular weight of the chitosan used for encapsulation may influence viability and encapsulation efficiency. Higher molecular weight chitosan (CS-HMW) beads typically form a denser, more compact structure, which could affect the diffusion of nutrients and the release of the encapsulated bioagent. Conversely, lower molecular weight chitosan forms more porous beads, allowing for better diffusion and potentially enhancing the viability and growth of *B. subtilis*. The observed successful encapsulation and viability of *B. subtilis* suggest that the chitosan beads, regardless of molecular weight, maintain their protective function while facilitating normal microbial development.

**Figure 5:**
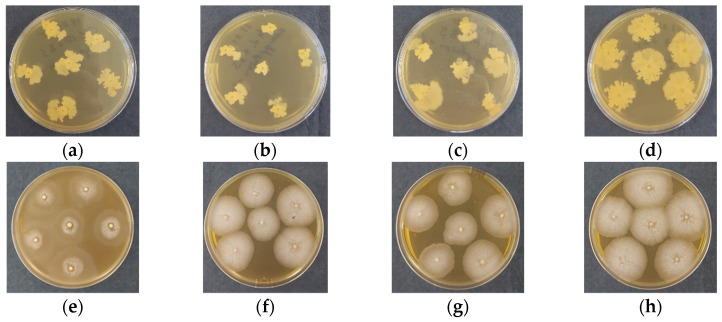
Digital images showing the growth of *B. subtilis* after 90 and 120 days of storage from: (**a**,**e**) COS/*B. subtilis*, (**b**,**f**) CS-LMW/*B. subtilis*, (**c**,**g**) CS-MMW/*B. subtilis* and (**d**,**h**) CS-HMW/*B. subtilis*. Panels (**a**–**d**) correspond to 90 days of storage, while panels (**e**–**h**) correspond to 120 days.

One of the main challenges in sustainable agriculture is preserving the activity of bioagents after encapsulation during long-term storage. The results demonstrated that *B. subtilis* exhibits high viability when encapsulated in chitosan gel beads. However, maintaining this viability over extended storage periods is even more critical. To confirm that encapsulation preserves viability during long-term storage, the survival of *B. subtilis* in chitosan gel beads with different molecular weights was tested after 90 and 120 days of storage in a desiccator at 4 °C. After this period, the samples were transferred to Petri dishes and incubated at 28 °C for seven days, after which bacterial growth was assessed. Digital photographs of the Petri dishes containing the beads are shown in Figure 5. For all samples, *B. subtilis* developed normally. Additionally, large growth zones were observed around the chitosan gel beads, which were formulated with chitosan of different molecular weights and contained *B. subtilis*. These results demonstrate that even after 120 days of storage, the encapsulated beneficial bacteria remained viable, grew, and reproduced normally. Thus, a novel, inexpensive, and simple method has been developed for preserving microorganisms.

### 2.4. Cultivation of Phytopathogens in the Presence of Chitosan Gel Beads with an Encapsulated Bioagent

The ability of chitosan gel beads encapsulating *Bacillus subtilis* to serve as novel and promising biocontrol agents was tested by evaluating their antifungal activity against the phytopathogens *Fusarium avenaceum* and *Rhizoctonia solani*. The selection of these pathogens for testing was based on their distinct characteristics. *Fusarium avenaceum* is an extremely aggressive pathogen that primarily affects the above-ground biomass of cereal crops, causing stem and basal damage. It exhibits abundant sporulation and is seed-borne [32]. In contrast, *Rhizoctonia* is a cosmopolitan, soil-borne pathogen with no species specificity. It does not form conidiospores and only rarely produces basidiospores. Instead, it develops persistent structures called sclerotia, which are highly resistant in the soil. *Rhizoctonia* often forms pathogenic complexes with other pathogens and nematodes, making it a particularly troublesome threat [33].

**Figure 6:**
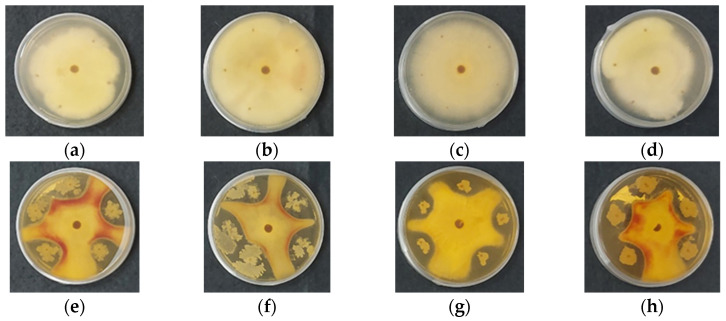
Digital images of coacervate gel beads of: (**a**) COS; (**b**) CS-LMW; (**c**) CS-MMW; (**d**) CS-HMW; (**e**) COS/*B. subtilis*; (**f**) CS-LMW/*B. subtilis*; (**g**) CS-MMW/*B. subtilis* and (**h**) CS-HMW/*B. subtilis* after 7 days of incubation in the presence of *Fusarium avenaceum*.

**Figure 7:**
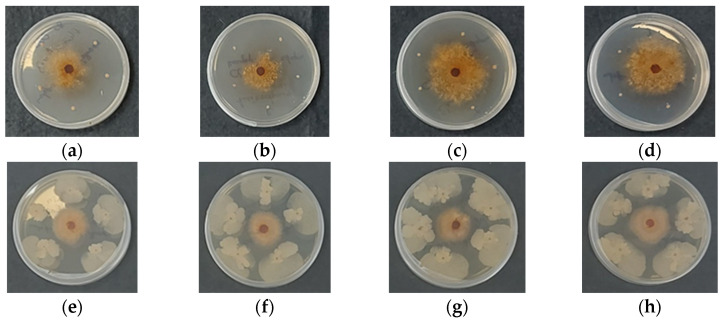
Digital images of coacervate gel beads of: (**a**) COS; (**b**) CS-LMW; (**c**) CS-MMW; (**d**) CS-HMW; (**e**) COS/*B. subtilis*; (**f**) CS-LMW/*B. subtilis*; (**g**) CS-MMW/*B. subtilis* and (**h**) CS-HMW/*B. subtilis* after 7 days of incubation in the presence of *Rhizoctonia solani*.

Chitosan gel beads containing *B. subtilis* were incubated for 7 days at 28 °C in Petri dishes, together with cells of the pathogenic microorganism. For comparison, the development of *Rhizoctonia* or *Fusarium* was also monitored in the presence of chitosan gel beads that did not contain *B. subtilis*. Toxicity tests against *B. subtilis* showed that the chitosan itself did not affect the development of the beneficial microorganism, nor did it impact the growth of the phytopathogens. In fact, *Fusarium* and *Rhizoctonia* developed normally in the presence of chitosan gel beads that did not contain *B. subtilis* (Figure 6 and Figure 7, panels a–d). However, the encapsulation of *B. subtilis* in the chitosan gel beads inhibited the development of both *Rhizoctonia* and *Fusarium*, regardless of the molecular weight of the chitosan used for bead preparation (Figure 6 and Figure 7, panels e–h).

In *Fusarium avenaceum*, rapid initial growth provides a competitive advantage in substrate colonization (Figure 6e–h). Similarly, spores incorporated within the chitosan coacervate beads develop rapidly, leading to substrate competition and the production of bioactive compounds that further inhibit pathogen proliferation. Substrate competition was intense and influenced by the polymer carrier. The increase in the molecular weight of the chitosan altered both the growth rate and colony morphology. Additionally, *F. avenaceum* exhibited red pigment release, which is likely associated with secondary metabolite production, particularly aurofusarin. This polyketide pigment is known to be influenced by environmental conditions, stress factors, and microbial competition. The presence of the biocontrol agent may have induced its production as part of the pathogen’s response to stress or competitive interactions.

In *Rhizoctonia solani*, the pathogen’s growth rate was significantly slower, giving the biocontrol agent a substantial advantage in substrate colonization (Figure 7e–h). The molecular weight of the polymeric carrier did not appear to influence interspecies interactions, as no discernible morphological differences were observed in the biocontrol agent’s colonies.

The observed antifungal activity of *B. subtilis* encapsulated in chitosan gel beads can be attributed to several potential mechanisms. First, *B. subtilis* is known to produce a range of antifungal compounds, such as lipopeptides that directly inhibit the growth of fungal pathogens [1,3]. These compounds are likely released from the beads into the surrounding environment, creating a hostile microenvironment for the pathogens. Second, *B. subtilis* may outcompete the fungi for nutrients and space, preventing their growth. Additionally, chitosan itself can serve as a nutrient source for *B. subtilis*, creating a favorable environment for the growth and development of the encapsulated microorganism. This mutualistic relationship between chitosan and *B. subtilis* could enhance the overall biocontrol effect. The presence of *B. subtilis* can also trigger induced resistance in plants through the production of systemic acquired resistance compounds, enhancing plant defense against fungal pathogens. The encapsulation in chitosan beads could also provide a controlled release of *B. subtilis*, allowing for sustained antifungal activity over time.

The results clearly show that *B. subtilis* encapsulated in chitosan gel beads effectively inhibits the growth of *Fusarium* and *Rhizoctonia*. Therefore, they underscore the potential of encapsulated *B. subtilis* as an effective biocontrol agent. It is worth noting that while the molecular weight of chitosan influences the structure and porosity of the beads, the encapsulation of *B. subtilis* appears to be effective against these pathogenic fungi across different chitosan formulations. Higher molecular weight chitosan beads, with their denser structure and smaller pores, may release *B. subtilis* more gradually, offering prolonged antifungal activity, while lower molecular weight chitosan beads, with their more porous structure, may facilitate quicker release and immediate activity.

## 3. Conclusions

This study highlights the effectiveness of chitosan gel beads, particularly when encapsulating *Bacillus subtilis*, as a biocontrol agent against fungal phytopathogens. The molecular weight of chitosan significantly affects the water retention and swelling behavior of the beads, with higher molecular weight chitosan retaining more water due to stronger intermolecular interactions. However, these beads experience greater shrinkage and weight loss during drying. Encapsulation effectively preserves the viability of *B. subtilis* spores, supporting their development and reproduction. Chitosan also provides a favorable environment for *B. subtilis*, enhancing its growth and activity. In particular, the beneficial bacterium *B. subtilis*, encapsulated in chitosan gel beads, exhibited normal growth and maintained its activity even after 90 and 120 days of long-term storage. Moreover, encapsulated *B. subtilis* exhibited significant antifungal activity against *Fusarium avenaceum* and *Rhizoctonia solani*, regardless of chitosan molecular weight. These findings demonstrate that chitosan not only serves as a carrier but also promotes the growth of *B. subtilis*, making this formulation a promising and eco-friendly biocontrol solution for plant protection.

## 4. Materials and Methods

### 4.1. Materials

Chitooligosaccharide (COS, 50 cPs, degree of deacetylation 92.1%) and chitosans of low (CS-LMW, max. 200 cPs), medium (CS-MMW, 200–400 cPs), and high (CS-HMW, min. 400 cPs) molecular weights (all with degree of deacetylation 91%) were purchased from Yantai Shang Tai Trading Co., Ltd., Beijing, China. Analytical-grade glacial acetic acid, sodium hydroxide, and buffer salts (sodium hydrogen carbonate, sodium carbonate, sodium hydrogen phosphate, and potassium dihydrogen phosphate) were supplied by Merck (Darmstadt, Germany). The following buffer solutions were prepared: pH 4.0 (CH_3_COOH/NaOH), pH 7.0 (KH_2_PO_4_/Na_2_HPO_4_), and pH 9.0 (NaHCO_3_/Na_2_CO_3_).

*Bacillus subtilis* microorganisms were obtained from the collection of Biodinamika Ltd. (Plovdiv, Bulgaria) and cultivated in Tryptic Soy Broth (TSB) medium (Biolife, Milan, Italy) at 28 °C on a rotary shaker at 197 rpm until complete sporulation. After 72 h (or 5 days, depending on experimental setup), bacterial spores were harvested by centrifugation at 6000 rpm and 4 °C for 15 min, followed by two washes with sterile distilled water. The final spore concentration was adjusted to 1 × 10^10^ spores/mL.

*Fusarium avenaceum* and *Rhizoctonia solani* strains were also obtained from the collection of Biodinamika Ltd. (Plovdiv, Bulgaria). Cultures were maintained on Potato Dextrose Agar (PDA) medium (Merck, Darmstadt, Germany). For inoculation, 5 mm diameter agar blocks containing established fungal cultures were placed at the center of PDA plates. The plates were incubated at 28 °C until the fungal mycelium had fully developed. In the case of *Fusarium avenaceum*, conidiospores were also formed.

### 4.2. Preparation of Chitosan Gel Beads and Encapsulation of Bacillus subtilis

Chitosan gel beads were prepared using a simple coacervation technique by extruding chitosan viscous solutions through a 20-gauge capillary into a 5% aqueous sodium hydroxide solution [30]. The concentrations of the chitosan solutions were as follows: 10% *w/v* COS in water and 2% *w/v* CS-LMW, 2% *w/v* CS-MMW, and 1.5% *w/v* CS-HMW in dilute acetic acid. The resulting spherical chitosan gel beads were kept in the alkaline precipitation bath for 1 h and then washed repeatedly with distilled water until the aqueous phase reached neutrality. A part of the beads was dried to a constant weight, while the rest were stored in water. For clarity, the freshly prepared gel beads will be referred to as “coacervate beads” to distinguish them from the “swollen” ones, which, after drying, were rehydrated to reach equilibrium swelling.

For the encapsulation of *Bacillus subtilis* into chitosan gel beads, the required amount of bacteria was added to the respective chitosan solutions: 10% *w/v* COS in water and 2% *w/v* CS-LMW, 2% *w/v* CS-MMW, and 1.5% *w/v* CS-HMW in dilute acetic acid, with 5 mL of the *Bacillus subtilis* suspension added per 1 g of chitosan. The obtained mixed solutions were extruded through a 19-gauge capillary into a 5% aqueous sodium hydroxide solution, kept for 1 h, and then washed repeatedly with distilled water until the aqueous phase reached neutrality.

### 4.3. Chitosan Gel Bead Characteriaztion

The average bead diameter was determined from a minimum of 10 measurements using a Bresser ADL-601P (Rhede, Germany) microscope. Bead surface morphology and cross-sectional SEM analysis were observed using a Jeol JSM-5510 (JEOL Co., Ltd., Tokyo, Japan) scanning electron microscope. Specimens were fixed on the sample holder, vacuum-coated with gold for 60 s in a Jeol JFC-1200 fine coater (JEOL Co., Ltd., Tokyo, Japan), and then examined. The mean bead diameter was also measured using ImageJ software version 1.54g.

The swelling degree was determined gravimetrically by immersing dry, pre-weighed beads into buffer solutions (pH 4, 7, and 9; ionic strength I = 0.1) at 25 °C. At regular time intervals, the excess of the buffer solution was gently removed, and the beads were then weighed. This process was repeated until a constant weight of the swollen beads was reached. After 24 h, the experiment was terminated as the swelling degree had decreased. The swelling degree (S, %) was calculated using the following equation:

(1) $$S,\;\%=\frac{\text{weight of swollen beads}-\text{weight of dry beads}}{\text{weight of dry beads}}\times 100$$

where the weights of the beads in the swollen state and the dry state are used, respectively.

### 4.4. Microbiological Tests

The viability of beneficial bacteria encapsulated in chitosan coacervate beads was evaluated based on their ability to form colonies on solid Tryptic Soy Agar (TSA) medium. Six chitosan gel beads were placed in each Petri dish on the surface of sterilized TSA and incubated at 28 °C. The growth dynamics of bacterial colonies were recorded after 48 h. As a control, chitosan gel beads without *Bacillus subtilis* were also tested for biological purity. Additionally, the viability of the encapsulated *Bacillus subtilis* within chitosan gel beads was assessed after 90 and 120 days of storage. Prior to the long-term storage study, the samples were stored in a desiccator at 4 °C. After 90 and 120 days, six chitosan coacervate beads were plated on solid TSA medium and incubated at 28 °C in a thermostat.

The biocontrol activity of chitosan gel beads encapsulating bacterial spores against fungal strains was assessed using a dual-culture technique on solid Triptic Soy Agar (TSA) (Biolife, Milan, Italy) medium. Agar plugs (5 mm in diameter) from 10-day-old fungal cultures were placed at the center of the TSA plates, and coacervate chitosan beads were arranged radially around the substrate. The dual cultures were incubated at 28 °C. Phytopathogen growth and the zones of suppression by the biocontrol agent were observed and recorded 7 days post-incubation.

## Figures and Tables

**Table 1:** Characteristics of the Prepared Chitosan Beads.

Chitosan Gel Beads	Average Weight, mg		Average Diameter, mm	
	Coacervate	Dry	Coacervate	Dry
COS	12.0 ± 0.3	1.03 ± 0.03	2.84 ± 0.27	1.30 ± 0.15
CS-LMW	8.6 ± 0.7	0.32 ± 0.01	2.57 ± 0.29	0.82 ± 0.12
CS-MMW	10.7 ± 0.2	0.32 ± 0.01	2.58 ± 0.26	0.81 ± 0.11
CS-HMW	9.7 ± 0.3	0.25 ± 0.02	2.76 ± 0.22	0.80 ± 0.18

**Table 2:** Swelling Degree of Chitosan Dry Beads as a Function of pH at 25 °C.

Chitosan Gel Beads	Swelling Degree, %		
	pH 4	pH 7	pH 9
COS	- ^1^	81.2	104.1
CS-LMW	- ^1^	83.4	110.5
CS-MMW	- ^1^	90.8	137.4
CS-HMW	- ^1^	136.7	179.1

^1^ All beads dissolve.

## Data Availability

The data are contained within this article.

## Supplementary Materials

The following supporting information can be downloaded at: https://www.mdpi.com/article/10.3390/gels11040302/s1, Figure S1: Cross-sectional SEM images of freeze-dried coacervate gel beads containing *Bacillus subtilis*: (a,b) COS/*B. subtilis*; (c,d) CS-LMW/*B. subtilis*; (e,f) CS-MMW/*B. subtilis*; (g,h) CS-HMW/*B. subtilis*.

## Abbreviations

The following abbreviations are used in this manuscript:

COS	Chitooligosaccharide
CS-LMW	Low Molecular Weight Chitosan
CS-MMW	Medium Molecular Weight Chitosan
CS-HMW	High Molecular Weight Chitosan
SEM	Scanning Electron Microscopy
