# Supplementary material for: Encapsulation of Bacillus subtilis in Chitosan Gel Beads for Eco-Friendly Crop Protection
Source: Gels. 2025 Apr 19;11(4):302. doi: 10.3390/gels11040302 (PMC12026960; doi:10.3390/gels11040302)
Supplement: Supplementary file 1 [file gels-11-00302-s001.zip › gels-3596013-supplementary.pdf]

gels

MDPI

Supplementary data

Encapsulation of *Bacillus subtilis* in Chitosan Gel Beads for Eco-Friendly Crop Protection

Vladimir Krastev [1], Nikoleta Stoyanova [1], Iliyana Valcheva [2], Donka Draganova [2], Mariya Spasova [1] and Olya Stoilova [1,*]

[1] Laboratory of Bioactive Polymers, Institute of Polymers, Bulgarian Academy of Sciences, Akad. G. Bonchev St., bl. 103A, 1113 Sofia, Bulgaria; v_krastev@polymer.bas.bg (V.K.); nstoyanova@polymer.bas.bg (N.S.); mspasova@polymer.bas.bg (M.S.); stoilova@polymer.bas.bg (O.S.)

[2] Biodinamika Ltd., 4000 Plovdiv, Bulgaria; donkadraganova@gmail.com (D.D.), valchevailiana1@gmail.com (I.V.)

* Correspondence: stoilova@polymer.bas.bg (O.S.)

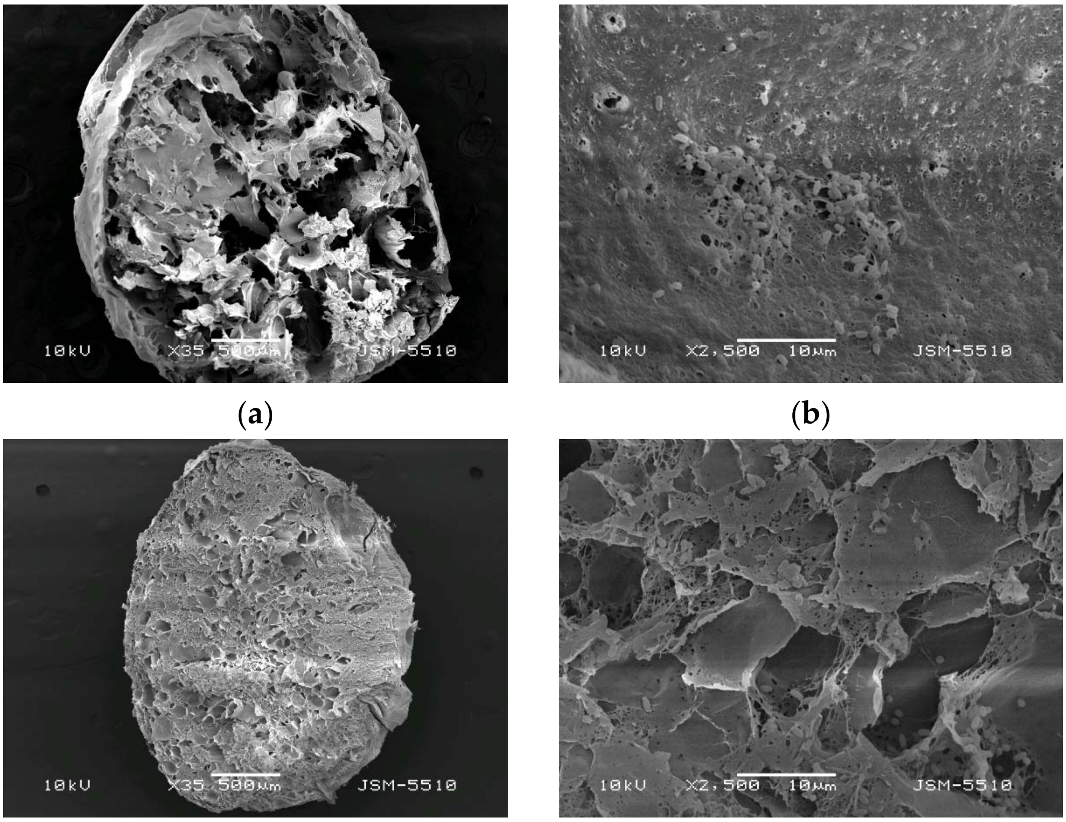

(**a**) (**b**) (**c**) (**d**)

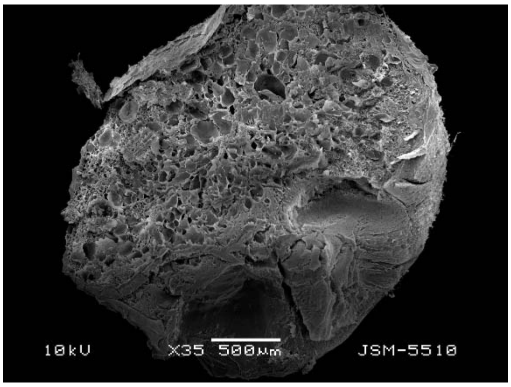

(**e**)

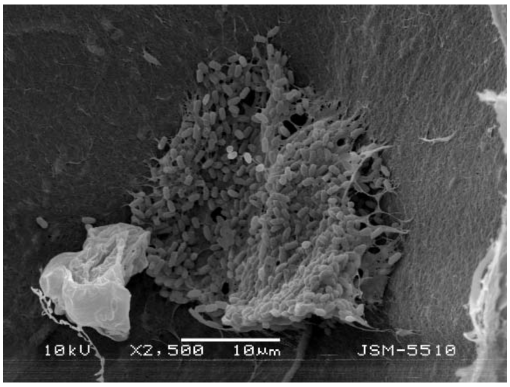

(**f**)

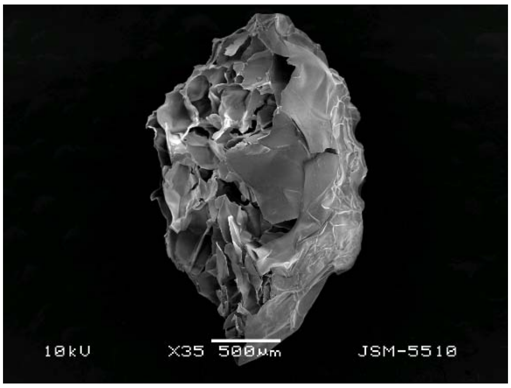

(**g**)

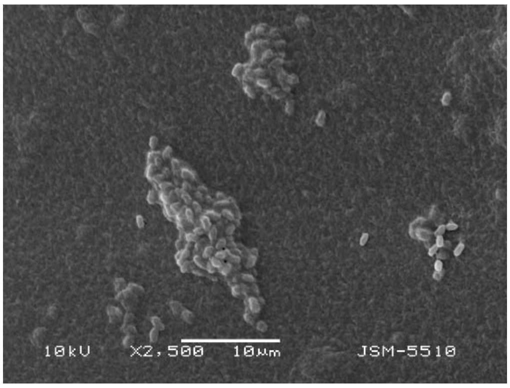

(**h**)

Figure S1. Cross-sectional SEM images of freeze-dried coacervate gel beads containing *Bacillus subtilis*: (a, b) COS/*B. subtilis*; (c, d) CS-LMW/*B. subtilis*; (e, f) CS-MMW/*B. subtilis*; (g, h) CS-HMW/*B. subtilis*.
